# Prevalence and new onset of depression and anxiety among participants with AMD in a European cohort

**DOI:** 10.1038/s41598-020-61706-8

**Published:** 2020-03-16

**Authors:** Jasmin Rezapour, Alexander K. Schuster, Stefan Nickels, Christina A. Korb, Hisham Elbaz, Tunde Peto, Matthias Michal, Thomas Münzel, Philipp S. Wild, Jochem König, Karl Lackner, Andreas Schulz, Norbert Pfeiffer, Manfred E. Beutel

**Affiliations:** 1 Department of Ophthalmology, University Medical Center of the Johannes Gutenberg-University Mainz, Mainz, 55131 Germany; 2 Hamilton Glaucoma Center, Shiley Eye Institute, Department of Ophthalmology, UC San Diego, La Jolla, CA 92037 United States; 3 NIHR Biomedical Research Center at Moorfields Eye Hospital NHS Foundation Trust and UCL Institute of Ophthalmology, London, EC1V 9EL United Kingdom; 4 Department of Ophthalmology, Otto-von-Guericke University, Magdeburg, 39106 Germany; 5 Queen’s University Belfast, Centre for Public Health, Belfast, BT7 1NN Northern Ireland United Kingdom; 6 Department of Psychosomatic Medicine and Psychotherapy, University Medical Center of the Johannes Gutenberg-University Mainz, Mainz, 55131 Germany; 7 Center for Cardiology I, University Medical Center of the Johannes Gutenberg-University Mainz, Mainz, 55131 Germany; 8 Preventive Cardiology and Preventive Medicine/Center for Cardiology, University Medical Center of the Johannes Gutenberg-University Mainz, Mainz, 55131 Germany; 9 Center for Thrombosis and Hemostasis (CTH), University Medical Center of the Johannes Gutenberg-University Mainz, Mainz, 55131 Germany; 10 German Center for Cardiovascular Research (DZHK), partner site Rhine-Main, Mainz, 55131 Germany; 11 Institute for Medical Biostatistics, Epidemiology and Informatics, University Medical Center of the Johannes Gutenberg-University Mainz, Mainz, 55131 Germany; 12 Institute for Clinical Chemistry and Laboratory Medicine, University Medical Center of the Johannes Gutenberg-University Mainz, Mainz, 55131 Germany

**Keywords:** Macular degeneration, Anxiety, Depression, Epidemiology

## Abstract

To investigate the prevalence and new onset of depression and anxiety among subjects with age-related macular degeneration (AMD) and its association with AMD in a large European cohort with relatively good visual acuity. 11,834 participants enrolled in the German population-based Gutenberg Health Study were studied. AMD was diagnosed by grading of fundus photographs. Depression and anxiety were assessed with the Patient Health Questionnaire and the Generalized Anxiety Disorder-2 Scale, respectively. Logistic regression analyses were performed and adjusted for several parameters. 1,089 (9.2%) participants were diagnosed having AMD. Prevalence of depression in AMD and non-AMD participants was 7.2% and 8.0%, respectively and prevalence of anxiety was 4.2% and 7.0%, respectively. New onset of depression and anxiety at 5-year follow-up in AMD subjects was 2.6% and 3.6%, respectively. AMD was not associated with depression (OR 0.93; CI 95% 0.70–1.20; p = 0.62). AMD was associated with less anxiety (OR 0.67; CI 95% 0.47–0.93; p = 0.02). This is the first study analyzing both prevalence and new onset of depression and anxiety in AMD subjects. AMD- and non-AMD participants had a similar prevalence and new onset of depression in our population-based sample. Participants without AMD had a higher prevalence of anxiety. AMD was not associated with depression.

## Introduction

Age-related macular degeneration (AMD) is a chronic, progressive eye disease that leads to central visual loss due to death of photoreceptors caused by loss of retinal pigment epithelium. Advanced stages may result in geographic atrophy and/or choroidal neovascularization in the central retina. Accounting for approximately 6% of blindness worldwide, AMD is the fourth most common cause of blindness^1^.

Age is one of the main risk factors for developing AMD^1^ and longer life expectancy in aging populations result in an increasing number of individuals with AMD^1,2^; an estimated 196 million individuals were affected in 2020 rising to a predicted 288 million in 2040^3^. In 2014, early and late AMD was detected in 11.9% and 0.2%, respectively, of individuals aged 35–74 years in Germany^4^. Treatment of “wet AMD” incorporates anti-VEGF injections that may slow down disease progression and stabilise vision^1,5^.

Recently, ophthalmologists have been advised to identify and consider mental distress in those with vision loss^6^ because advanced vision loss has a major impact on a person’s quality of life. An inability to continue work and hobbies, and decreased mobility may adversely affect social integration and can be associated with depression. While depression is one of the leading contributors to the global disease burden, it is often accompanied by anxiety^7^. Accordingly, the prevalence of depression and anxiety in AMD patients has been subject of several studies, however reported results have been inconsistent.

A European study found that poor visual acuity, but not the presence of AMD was positively associated with depression, whereas anxiety was unrelated^8^. In a systematic review, the prevalence of depression and anxiety was in the range of 15.7–44.0% and 9.6–30.0%, respectively, for patients with AMD. Case-control studies analysed in this review found patients with AMD were more likely to have symptoms of depression, but not of anxiety, compared to those without AMD. However, due to the heterogeneity of studies (sample size, measurement methods, study design etc.) and reporting (lack of relevant clinical data, cut-off scores etc.) a formal meta-analysis was not performed^9^. In contrast, Jonas *et al*. and Sun *et al*. found no association between AMD and depression in large cohort studies, adjusted for a wide range of socioeconomic variables and systemic diseases^10,11^.

The large and ever increasing number of patients affected by AMD in the context of an aging population reveals the importance of studying possible associated mental health conditions. Findings to date are contradictory due to lack of large-scale and prospective community-based studies. Most of the hospital-based case-control studies are limited by small sample sizes and strict inclusion criteria, such as AMD patients with advanced disease and significant vision loss.

To the best of our knowledge, there is no population-based European cohort study investigating the prevalence and new onset of depression and anxiety among subjects with AMD and the association between both mental conditions and AMD. The current study uses cross-sectional and longitudinal data from the Gutenberg Health Study (GHS) to determine the prevalence and new onset of depression and anxiety among subjects with AMD and to analyze the association between both mental conditions and AMD.

## Methods

The Gutenberg Health Study (GHS) is an ongoing population-based, interdisciplinary, prospective, observational single-center cohort study in the Rhein-Main Region in western mid-Germany. It was approved by the Ethics Commission of the State Chamber of Physicians of Rhineland-Palatinate (reference no. 837.020.07, original vote: 22.3.2007, latest update: 20.10.2015). According to the Declaration of Helsinki, written informed consent was obtained from all subjects before entering the study. The population sample was randomly drawn via local residents’ registration offices and equally stratified by sex and residence (urban/rural) for each decade of age. Patients selected were contacted by letter and if they did not respond, they were contacted by the recruitment team by telephone. Exclusion criteria were physical and mental disability precluding a visit to the study center on their own and an insufficient knowledge of the German language. A detailed description of the study design has been published elsewhere^12^.

### Study sample

Cross-sectional data analyses were based on 11,834 participants (out of 15,010 participants) aged 35 to 74 years at the baseline of the Gutenberg Health Study (GHS), for whom ophthalmological findings, depression and anxiety scores were available. Longitudinal analyses were based on data from 8,098 and 9,039 participants with available depression and anxiety measurements, respectively, with 5-year follow-up examinations carried out between April 2012 and April 2017 with an overall participation rate of 82.8% (see Beutel *et al*. 2018 for details)^13^. The 5-year follow-up response proportion for subjects without depression at baseline and with and without AMD at baseline was 79.4% and 84.8%, respectively, and for subjects with depression at baseline and with and without AMD 82.0% and 77.8%, respectively. The 5-year response rate for subjects without anxiety at baseline with and without AMD was 79.9% and 84.6%, respectively, and for subjects with anxiety at baseline 78.3% and 80.9%, respectively.

### Materials and assessment

The baseline examination was carried out between 2007 and 2012, lasted 5 hours and included evaluation of prevalent classical cardiovascular risk factors and clinical variables, laboratory examinations from a venous blood sample, blood pressure and anthropometric measurements, as well as ophthalmological examinations and computer-assisted personal interviews^14^.

### Ophthalmological examinations

The ophthalmic assessment was described in detail by Höhn *et al*.^15^. In brief, the ophthalmic examinations included objective refraction and distance-corrected visual acuity (DCVA) (Humphrey Automated Refractor/Keratometer [HARK] 599; Carl Zeiss Meditec AG, Jena, Germany). First autorefraction was obtained, followed by corrected visual acuity with the built-in Snellen charts, ranging from 1.3 to −0.3 logMar. In case of visual acuity below 1.3 logMar, testing was performed with a visual acuity chart at a distance of one meter and if this was not possible, counting fingers, hand movements, light perception and no light perception were tested^15^. Visual field screening, intraocular pressure measurement (IOP) (Nidek NT-2000; Nidek, Co., Gamagori, Japan) and non-mydriatic fundus photography (Visucam PRO NM™, Carl Zeiss Meditec AG, Jena, Germany) also was obtained.

For AMD grading, fundus images were taken with a non-mydriatic fundus camera (Visucam PRO NM™, Carl Zeiss AG, Jena, Germany) under mesopic light conditions. Three photographs of each eye were taken at 30° centered on the macula. The fundus images were assessed by a German board certified consultant of ophthalmology (HEB) using the Irfan View 4.41 program and a 24” high resolution monitor. Ambiguous cases were discussed with a senior grader from the reading center of Moorfields Eye Hospital (TP). Based on the Rotterdam Eye Study classification the presence or absence of AMD signs within a radius of two disc diameters from the fovea were graded^16,17^. Age-related changes in the macular region were categorized as early (stages 1a, 1b, 2a, 2b, 3) and late (stages 4a and 4b) maculopathy. Maculopathy unrelated to AMD, for example due to vascular diseases or diabetes, was categorized as stage 5. Small, hard drusen were excluded from the AMD grading according to the classification of the International AMD Epidemiology Study Group^18^. AMD grading is described in detail by Korb *et al*.^4^.

Self-reported eye diseases were assessed by interview during the eye examination. Participants were asked if they had been diagnosed with glaucoma, cataract, macular degeneration or corneal diseases.

### Questionnaires

All questionnaires were assessed at baseline examination and at 5-year follow-up examination.

### Depression

Depression was assessed with the Patient Health Questionnaire (PHQ-9), which evaluates how often subjects have been bothered by any of the nine diagnostic criteria of Major depression over the last two weeks. The total points for each of the items were summed to create a score between 0 and 27 points. Depression was defined as a sum score of ≥10. A prior study showed a sensitivity of 81% and a specificity of 82% for any depressive disorder^19^. In addition, depressive symptoms were classified as “minimal” (score 5 to 9), “mild” (score 10 to 14), “moderately severe” (score 15 to 19) and “severe” (score >20)^20^.

### Generalized anxiety

Generalized anxiety was measured with the two screening items of the short form of the GAD-7 (Generalized Anxiety Disorder [GAD]−7 Scale), a screening tool for all anxiety disorders. With lower sensitivity and specificity values the questionnaire can also be used to screen for panic disorder, social anxiety and post-traumatic stress disorder. Core anxiety symptoms are represented by the first two screening items of the GAD-7. Participants rated “Feeling nervous, anxious or on edge” and “Not being able to stop or control worrying” as 0 = “not at all”, 1 = “several days”, 2 = “over half the days”, and 3 = “nearly every day” over the last two weeks. The total points for both items were summed and ranged from 0 to 6^21^. Generalized anxiety (GAD-2) was defined as a sum score of ≥3, corresponding to a sensitivity of 86% and a specificity of 83%^21^.

### Type D Personality

Type D Personality comprises both high negative affectivity and social inhibition and is related to poor cardiac health and suicidal ideation^22–24^. Individuals with a high negative affectivity often feel dysphoria, anxiety and irritability and those with social inhibition often feel inhibited and insecure in public. We assessed Type D Personality in our study design to include it in our regression analyses as a potential confounder for depression and anxiety.

The German version of the DS-14 was used to detect Type D Personality^25^. The DS-14 consists of two subscales with seven items describing examples of negative affectivity (NA) and seven items describing examples of social inhibition (SI), which were answered on a 5-point-Likert scale from 0 (false) to 4 (true). Type D personality is defined as a cutoff ≥10 on both subscales^24^.

### Panic disorder

Panic disorder was measured with the PHQ panic module. Caseness for panic disorder was defined if at least two of four PHQ panic questions were answered with “yes” (sensitivity 91%, specificity 88%)^26^.

### Social support

The Brief Social Support Scale assessed emotional and tangible support (three items per scale) with good reliability (total scale α = 0.86). Items were rated from 1 = never, 2 = occasionally, 3 = mostly to 4 = always available^27^.

### Loneliness

Loneliness was assessed by the item “I am frequently alone/have few contacts” rated as 0 = no, does not apply; 1 = yes it applies, but I do not suffer from it; 2 = yes, it applies, and I suffer slightly; 3 = yes, it applies, and I suffer moderately; 4 = yes, it applies, and I suffer strongly^28^.

### Computer-assisted personal Interview

During the computer-assisted personal interview, subjects were asked in person if they had a history of any depressive or anxiety condition. According to Lampert and Kroll, socioeconomic status (SES) was defined by education, income and job position, with a range from 3 to 21, while 3 indicated the lowest SES and 21 the highest SES^29^. For statistical analyses, three groups were defined including low SES (3–8 points), medium SES (9–14 points) and high SES (15–21 points)^29^. Participants were asked if they currently lived in a partnership and if they were employed. Furthermore, subjects were asked to classify their general health condition into four categories (excellent = 1, good=2, fair=3 and poor=4), and self-reported presence of the following general diseases was collected from the personal interview: arterial hypertension, myocardial infarction, stroke, diabetes mellitus, chronic obstructive pulmonary disease (COPD), and bronchial asthma.

### Statistical analyses

Descriptive analyses were performed as absolute and relative proportions for categorical data, mean and standard deviation for continuous variables with approximately normal distribution and median with interquartile range if not fulfilling this criterion. Baseline parameters were reported for the total study group and separately for the presence of AMD. Prevalence rates of depression and depressive symptoms and generalized anxiety were given for participants with and without AMD and according to different age groups and visual acuity.

New onset of depression and anxiety was defined as exceeding the respective cut-off scores (PHQ-9 ≥ 10; GAD-2 ≥ 3) at follow-up, without having increased depression and anxiety scores at baseline.

A sensitivity analysis was performed to evaluate AMD subjects with advanced disease. Prevalence of depression and anxiety was computed for subjects with diagnosed AMD in relation to AMD stage and with a visual acuity >0.3 logMar in the a) worse eye and b) best eye. As an additional sensitivity analysis we reported depression and anxiety prevalence after excluding participants under treatment with antidepressants or anxiolytics. Comparisons between groups were done with chi-square-test for categorical variables and with Mann-Whitney U-test and t-test for continuous variables.

To determine the relationship between AMD and depression (caseness: PHQ-9 sum score <10 vs. PHQ-9 sum score ≥10) and generalized anxiety (caseness: GAD-2 sum score <3 vs. GAD-2 sum score ≥3) we performed logistic regression analysis with depression and anxiety as the dependent variable and AMD as the independent variable. In model 1, AMD was included as the independent variable, adjusted for age, sex and socio-economic status. In model 2, we also adjusted for self-reported ocular diseases (including cataract, glaucoma, corneal disease), systemic comorbidities (arterial hypertension, myocardial infarction, stroke, diabetes mellitus, chronic obstructive pulmonary disease, bronchial asthma), visual acuity of the best eye, mental health (Type D personality, loneliness, social support) and general health status.

All p-values should be regarded as continuous parameters that reflect the level of statistical evidence and are therefore reported exactly. Statistical analysis was carried out using R version 3.3.1^30^.

### Ethics approval and consent to participate

The Gutenberg Health Study was approved by the ethics committee (Ethics Commission of the State Chamber of Physicians of Rhineland-Palatinate, reference no. 837.020.07, original vote: 22.3.2007, latest update: 20.10.2015). According to the Declaration of Helsinki, written informed consent was obtained from all subjects before entering the study.

## Results

Results from a total of 11,834 participants were analyzed (5,876 men and 5,958 women; mean age 54.4 ± 11.0 years), of whom 1,089 (9.2%) were diagnosed as having AMD. Early AMD (stages 1–3) was present in 95.2% (1037) of the participants with AMD, stage 1a, 21.1% (230), stage 1b, 49.0% (534), stage 2a, 13.0% (142), stage 2b, 3% (39), stage 3, 8.5% (92), late AMD (stages 4a + 4b) was present in 4.8% (52) of the participants, stage 4a, 4.2% (46), stage 4b, 0.6% (6). Individuals in the AMD group were older than those in the control group (61.8 ± 9.9 years vs. 53.6 ± 10.8, respectively, p < 0.0001). Subjects with AMD had a lower SES (12.92 ± 4.55 vs. 13.12 ± 4.42, p < 0.0001) and were less frequently employed (38.8% vs. 65.1%, p = 0.0001). Social parameters including being in a partnership, receiving social support and having a sense of loneliness as well as mental health conditions such as Type D Personality and panic disorder were distributed similarly in both groups. There was a significant difference in visual acuity (VA) between both groups. However visual acuity was relatively good, 0.1 logMar and 0 logMar, respectively. for participants with and without AMD (p < 0.001). Demographic data of the total study cohort and stratified for the presence of AMD/no AMD are presented in Table 1.

**Table 1:** Characteristics of participants with (N = 1,089) and without (N = 10,745) AMD, with available psychiatric measurements at baseline in the Gutenberg Health Study 2007–2012.

	All (N = 11834)	No AMD (N = 10745)	AMD (N = 1089)	p-value
Age (years)	54.4 ± 11.0	53.6 ± 10.8	61.8 ± 9.9	<0.0001
Sex (women)	50.3% (5958)	50.7% (5446)	47.0% (512)	0.02
*Systemic diseases*				
Arterial Hypertension (yes)	48.6% (5744)	46.9% (5037)	65.0% (707)	<0.0001
Myocardial infarction (yes)	2.5% (294)	2.3% (248)	4.2% (46)	0.00031
Stroke (yes)	1.7% (204)	1.6% (174)	2.8% (30)	0.01
Diabetes mellitus	8.5% (1000)	8.0% (855)	13.3% (145)	<0.0001
COPD (yes)	4.9% (579)	4.8% (517)	5.7% (62)	0.21
Bronchial asthma (yes)	2.8% (337)	2.8% (305)	2.9% (32)	0.85
*Ocular characteristics*				
Visual acuity (logMar)	0 (0/0.10)	0 (0/0.10)	0.10 (0/0.22)	<0.0001
Glaucoma (self-reported)	2% (253)	1.9% (208)	4.1% (45)	<0.0001
Corneal disease (self-reported)	2% (242)	1.9% (208)	3.1% (34)	0.01
Cataract (self-reported)	0.6% (66)	0.5% (54)	1.1% (12)	0.02
*Social parameter*				
SES	13.02 ± 4.44	13.12 ± 4.42	12.02 ± 4.55	<0.0001
Employed	62.7% (7391)	65.1% (6972)	38.8% (419)	<0.0001
Partnership	81.3% (9613)	81.4% (8740)	80.2% (873)	0.37
Social support	21.00 (18.00/24.00)	21.00 (18.00/24.00)	21.00 (18.00/24.00)	0.38
General health status (Glhs): Excellent	12.9% (1530)	13.0% (1401)	11.9% (129)	0.30
Glhs: Good	67.1% (7939)	67.3% (7227)	65.6% (712)	0.25
Glhs: Fair	17.2% (2031)	17.0% (1823)	19.2% (208)	0.08
Glhs: Poor	2.8% (326)	2.7% (289)	3.4% (37)	0.17
*Mental health*				
Type D Personality	24.3% (2867)	24.2% (2595)	25.1% (272)	0.53
Panic disorder	4.8% (553)	4.8% (500)	5.1% (53)	0.70
Antidepressants	5.6% (655)	5.5% (586)	6.4% (69)	0.24
Anxiolytics	0.9% (104)	0.8% (84)	1.9% (20)	0.002

Abbreviations: COPD (chronic obstructive pulmonary disease), Glhs (general health status), SES (socioeconomic status).

### Depression

The prevalence of depression in the study population overall was 8.0% (n = 942) and was similar between AMD and non-AMD participants (AMD: 7.2%, non-AMD: 8.0%). New onset of depression (AMD: 2.6%, non-AMD: 4.5%) was more frequent in non-AMD subjects (Table 2). In both groups, most of the subjects were diagnosed with minimal depressive symptoms. There was a negligible prevalence of severe depressive symptoms in both groups (AMD: 0.3%, non-AMD: 0.5%) (Table 2). Regarding age, in both groups, AMD and non-AMD, the prevalence of depression was highest in participants between 45 and 54 years (Table 3).

**Table 2:** Prevalence and new onset of depression and generalized anxiety in participants with and without AMD in the Gutenberg Health Study.

	All (N = 11834)	No AMD (N = 10745)	AMD (N = 1089)
PHQ-9	3.00 (2.00/6.00)	3.00 (2.00/6.00)	3.00 (2.00/5.62)
PHQ-9 ≥ 10	8.0% (942)	8.0% (864)	7.2% (78)
PHQ-9 (Minimal)	27.6% (3264)	27.6% (2963)	27.6% (301)
PHQ-9 (Mild)	6.0% (712)	6.0% (648)	5.9% (64)
PHQ-9 (Moderately)	1.4% (171)	1.5% (160)	1.0% (11)
PHQ-9 (Severe)	0.5% (59)	0.5% (56)	0.30% (3)
New onset PHQ-9 ≥ 10	4.4% (355)	4.5% (337)	2.6% (18)
GAD-2	1.00 (0/1.00)	1.00 (0/1.00)	0 (0/1.00)
GAD-2 ≥ 3	6.7% (791)	7.0% (745)	4.2% (46)
New onset GAD-2 ≥ 3	4.7% (421)	4.8% (393)	3.6% (28)

Abbreviations: AMD (age-related macular degeneration), PHQ-9 (Patient Health Questionnaire), GAD-2 (Generalized Anxiety Disorder Scale).

PHQ-9 ≥ 10 defined as caseness for depression, GAD-2 ≥ 3 defined as caseness for generalized anxiety.

**Table 3:** Depression and anxiety prevalence in AMD participants by age and visual acuity.

AMD			
Age in years (n)	Mean visual acuity (logMar)	PHQ-9 ≥ 10	GAD-2 ≥ 3
35–44 (93)	0.1	8.6%	3.2%
45–54 (157)	0.1	11.5%	7.6%
55–64 (318)	0.1	8.5%	4.7%
65–74 (521)	0.2	4.8%	3.1%
**Non-AMD**			
35–44 (2664)	0.0	8.5%	8.6%
45–54 (3115)	0.0	10.0%	8.4%
55–64 (2802)	0.1	7.7%	6.1%
65–74 (2164)	0.1	5.2%	4.1%

Abbreviations: AMD (age-related macular degeneration), GAD-2 (Generalized Anxiety Disorder Scale), PHQ-9 (Patient Health Questionnaire).

PHQ-9 ≥ 10 defined as caseness for depression.

GAD-2 ≥ 3 defined as caseness for generalized anxiety.

To analyze an association between AMD and depression we performed logistic regression analysis. After adjusting for age, sex and SES we found no association between AMD and prevalence of depression (OR 1.03; CI 95% 0.80–1.31; p = 0.84). Controlling additionally for ocular diseases, systemic diseases, visual acuity of the best eye, Type D personality, social support, loneliness and general health status there was no association between AMD and depression (OR 0.93; CI 95% 0.70–1.20; p = 0.62) (Table 4 and Supplementary Table S1). Sex, Type D personality, loneliness, social support and general health status were significantly associated with depression (Table 4).

**Table 4:** Associations (multivariate logistic regression analysis) of AMD with depression in the Gutenberg Health Study (GHS) and significantly associated parameters.

PHQ-9 ≥ 10 (N = 11780)	*Model 1*		
	OR	CI	p-value
AMD	1.03		
Sex (Women)	1.48	1.29–1.70	<0.0001
Age [5 years]	0.89	0.86–0.92	<0.0001
SES	0.93	0.92–0.95	<0.0001
**PHQ-9 ≥ 10 (N = 11421)**	***Model 2***		
	**OR**	**CI**	**p-value**
AMD	0.93	0.70–1.20	0.62
Sex (Women)	1.36	1.15–1.59	0.01
Age [5 years]	0.90	0.87–0.94	<0.0001
SES	0.99	0.97–1.01	0.46
Visual acuity best eye	0.51	0.20–1.25	0.15
Type D personality	3.02	2.58–3.53	<0.0001
Social support	0.91	0.90–0.93	<0.0001
Loneliness	2.98	2.48–3.57	<0.0001
General Health status (from good to poor)	3.41	3.05–3.83	<0.0001

Abbreviations: CI (95% confidence Interval), OR (Odds ratio), PHQ-9 (Patient Health Questionnaire), SES (socioeconomic status).

PHQ-9 ≥ 10 defined as caseness for depression.

Model 1: Logistic regression analysis adjusted for age, sex, socio-economic status.

Model 2*: Additionally, adjusted for systemic comorbidities, self-reported ocular diseases, visual acuity of the best eye, mental health (Type D personality, loneliness, social support) and general health status.

*not significantly associated parameters (systemic comorbidities, ocular diseases) are not presented in this table. Model 2 with full list of covariates can be found in the Supplementary Table S1.

### Anxiety

The prevalence of generalized anxiety in all study participants was 6.7% (n = 791). Subjects without AMD were more often anxious than those with AMD (7.0% vs. 4.2%) at baseline. New-onset of anxiety was present in 3.6% and 4.8% for AMD and non-AMD subjects, respectively (Table 2). Participants with AMD used more often anxiolytics (1.9%) than those without AMD (0.8%). (Table 1). The prevalence of generalized anxiety was highest in subjects between 45 and 54 years in the AMD group and highest in subjects between 35 and 44 years in the non-AMD group (Table 3).

To analyze the association between AMD and anxiety we performed logistic regression analysis. After adjusting for age, sex and SES there was no association between AMD and prevalent anxiety (OR 0.74; CI 95% 0.54–1.00; p = 0.06). Controlling additionally for ocular diseases, systemic diseases, visual acuity of the best eye, Type D personality, social support, loneliness and general health status we found an association between AMD and lower anxiety (OR 0.67; CI 95% 0.47–0.93; p = 0.02) (Table 5 and Supplementary Table S2). Sex, Type D personality, loneliness, social support and general health status were significantly related to anxiety (Table 5).

**Table 5:** Associations (multivariate logistic regression analysis) of AMD with generalized anxiety in the Gutenberg Health Study (GHS) and significantly associated parameters.

GAD-2 ≥ 3 (N = 11719)	*Model 1*		
	OR	CI	p-value
AMD	0.74	0.54–1.00	0.06
Sex (Women)	1.59	1.38–1.86	<0.0001
Age [5 years]	0.87	0.84–0.89	<0.0001
SES	0.96	0.94–0.98	<0.0001
**GAD-2 ≥ 3 (N = 11409)**	***Model 2***		
	**OR**	**CI**	**p-value**
AMD	0.67	0.47–0.93	0.02
Sex (Women)	1.48	1.26–1.75	<0.0001
Age [5 years]	0.89	0.85–0.93	<0.0001
SES	1.01	0.99–1.03	0.21
Visual acuity best eye	0.47	0.17–1.24	0.13
Type D personality	2.79	2.37–3.29	<0.0001
Social support	0.94	0.93–0.96	<0.0001
Loneliness	2.36	1.94–2.86	<0.0001
General Health status (from good to poor)	2.61	2.33–2.94	<0.0001

Abbreviations: CI (95% confidence Interval), GAD-2 (Generalized Anxiety Disorder Scale), OR (Odds ratio), SES (socioeconomic status).

GAD-2 ≥ 3 defined as caseness for generalized anxiety.

Model 1: Logistic regression analysis adjusted for age, sex, socio-economic status.

Model 2*: Additionally, adjusted for systemic comorbidities, self-reported ocular diseases, visual acuity of the best eye, mental health (Type D personality, loneliness, social support) and general health status.

*not significantly associated parameters (systemic comorbidities, ocular diseases) are not presented in this table. Model 2 with full list of covariates can be found in the Supplementary Table S2.

### Sensitivity analysis

To identify the prevalence of depression and anxiety in a subsample with a reduced visual acuity we analyzed all participants with a visual acuity >0.3 logMar of the a) worse eye (Table 6) and b) the best eye (Table 7). There was no difference in AMD participants with (16%) and without (17.8%) prevalent depression (p = 0.79) in the worse eye subsample (Table 6) and with (29.2%) and without (28.4%) prevalent depression (p = 1.00) in the best eye subsample (Table 7). We stratified the subsample into participants with early and late AMD. Prevalence of depression and no depression remained similar in both AMD groups (Tables 6 and 7). Prevalence of anxiety (13.3%) and no anxiety (18.0%) was also similar (p = 0.30) in participants with AMD and a visual acuity >0.3 logMar in the worse (Table 6) and in the best eye (33.3% and 28.2%, respectively; p = 0.62) (Table 7). Prevalence of anxiety was similar in early and late AMD in both subsamples (Tables 6 and 7).

**Table 6:** Prevalence of depression and anxiety in a subsample with visual acuity >0.3 logMar of the worse eye.

Total	Depression (no) (N = 1370)	Depression (yes) (N = 106)	p-value^a^
AMD	17.8% (244)	16% (17)	0.79
Early AMD (1–3)	13.4% (184)	10% (11)	0.46
Late AMD (4)	2.2% (30)	4.7% (5)	0.10
**Total**	**Anxiety (no) (N = 1376)**	**Anxiety (yes) (N = 83)**	
AMD	18% (248)	13.3% (11)	0.30
Early AMD (1–3)	13.4% (185)	9.6% (8)	0.40
Late AMD (4)	2.3% (32)	3.6% (3)	0.45

^a^chi-square test

Abbreviations: AMD (age-related macular degeneration).

**Table 7:** Prevalence of depression  and anxiety in a subsample with visual acuity >0.3 logMar of the best eye.

Total	Depression (no) (N = 261)	Depression (yes) (N = 24)	p-value^a^
AMD	28.4% (74)	29.2%(7)	1.00
Early AMD (1–3)	22.6%% (59)	20.8% (5)	1.00
Late AMD (4)	5.7% (15)	8.3% (2)	0.64
**Total**	**Anxiety (no) (N = 259)**	**Anxiety (yes) (N = 21)**	
AMD	28.2% (73)	33.3% (7)	0.62
Early AMD (1–3)	22.0% (57)	28.6% (6)	0.59
Late AMD (4)	6.2% (16)	4.8% (1)	1.00

^a^chi-square test.

Abbreviations: AMD (age-related macular degeneration).

To exclude treatment for depression and/or anxiety as confounding factors we conducted a second sensitivity analysis. After excluding all participants treated with antidepressants or anxiolytics at time of the study, prevalence and new onset of depression and anxiety was similar to our previous findings. Prevalence of depression and anxiety was 5.9% and 3.6%, respectively, in the AMD group and 6.1% and 5.6% in the non-AMD group (Table 8). AMD was not associated with depression (OR 1.05; CI 95% 0.75–1.45; p = 0.75) or anxiety (OR 0.75; CI 95% 0.50–1.08; p = 0.14) in the regression analysis.

**Table 8:** Prevalence and new onset of depression and generalized anxiety in participants with and without AMD after excluding participants under treatment with antidepressants or anxiolytics.

	All (N = 10683)	No AMD (N = 9715)	AMD (N = 968)
PHQ-9 ≥ 10	6.1% (651)	6.1% (594)	5.9% (57)
New onset PHQ-9 ≥ 10	4.1% (319)	4.3% (304)	2.3% (15)
GAD-2 ≥ 3	5.4% (575)	5.6% (540)	3.6% (35)
New onset GAD-2 ≥ 3	3.9% (325)	4.0% (306)	2.6% (19)

Abbreviations: AMD (age-related macular degeneration), PHQ-9 (Patient Health Questionnaire), GAD-2 (Generalized Anxiety Disorder Scale).

PHQ-9 ≥ 10 defined as caseness for depression, GAD-2 ≥ 3 defined as caseness for generalized anxiety.

Comparing characteristics of AMD participants with (n = 78) and without depression (n = 1011), those with prevalent depression were younger (58.8 ± 10.1 years vs. 62.0 ± 9.9 years; p = 0.0089), lived less often in a partnership (68% vs. 81.2%; p = 0.07), received less social support (16 vs. 21; p < 0.0001), were more often lonely (36.8% vs. 8.6%; p < 0.0001), had more often a poor general health status (17% vs. 2%; p < 0.0001) and were more often diagnosed with Type D pesonality (70% vs. 21.7%; p < 0.0001), panic disorder (28% vs. 4%; p < 0.0001) and generalized anxiety (39% vs. 2%; p < 0.0001). More AMD participants with prevalent depression used antidepressant medication (20% vs. 5%; p < 0.0001). There was no significant difference in visual acuity for AMD subjects with and without prevalent depression (both 0.1 logMar; p = 0.7) (Table 9).

**Table 9:** Study characteristics of AMD participants with and without depression.

	No depression (1011)	Depression (78)	p-value
Age (years)	62.0 ± 9.9	58.8 ± 10.1	0.0089
Sex (women)	46.8% (473)	50% (39)	0.64
*Ocular parameter* Visual acuity (logMar)	0.1 (0/0.22)	0.1 (0/0.22)	0.7
*Social parameter*			
SES	12.06 ± 4.56	11.50 ± 4.38	0.28
Employed	38.5% (386)	42.3% (33)	0.55
Partnership	81.2% (820)	68% (53)	0.01
Social support	21.00 (18.00/24.00)	16.00 (13.00/20.00)	<0.0001
Loneliness	8.6% (86)	36.8% (28)	<0.0001
General health status (Ghls): Excellent	12.4% (125)	5.1% (4)	0.07
Ghls: Good	67.7% (682)	38.5% (30)	<0.0001
Ghls: Fair	17.6% (177)	40% (31)	<0.0001
Ghls: Poor	2.4% (24)	16.7% (13)	<0.0001
*Mental health*			
Generalized anxiety	1.6% (16)	38.5% (30)	<0.0001
Type D Personality	21.7% (219)	69.7% (53)	<0.0001
Panic disorder	3.5% (34)	27.9% (19)	<0.0001
Antidepressants	5.4% (54)	19.5% (15)	<0.0001
Anxiolytics	1.9% (17)	3.9% (3)	0.17

Abbreviations: Glhs (general health status), SES (socioeconomic status).

For AMD participants with prevalent generalized anxiety different study characteristics were similar to those of AMD participants with and without prevalent depression. However AMD participants with and without generalized anxiety had similar age (59.5 ± 9.8 vs. 61.8 ± 9.9; p = 0.12) and were also more often diagnosed with prevalent depression (65% vs. 5%; p < 0.0001) (Table 10).

**Table 10:** Study characteristics of AMD participants with and without anxiety.

	No anxiety (1037)	Anxiety (46)	p-value
Age (years)	61.8 ± 9.9	59.5 ± 9.8	0.12
Sex (women)	46.9% (486)	47.8% (22)	1.00
*Ocular parameter* Visual acuity (logMar)	0.1 (0/0.22)	0.13 (0/0.26)	0.34
*Social parameter*			
SES	12.03 ± 4.55	12.35 ± 4.53	0.65
Employed	38.6% (397)	45.7% (21)	0.36
Partnership	80.5% (834)	71.7% (33)	0.18
Social support	21 (18.00/24.00)	18.00 (13.00/21.00)	<0.0001
Loneliness	9.5% (98)	34.1% (15)	<0.0001
General health status (Ghls): Excellent	12.5% (129)	0% (0)	0.001
Ghls: Good	66.7% (690)	41.3% (19)	0.001
Ghls: Fair	17.8% (184)	45.7% (21)	<0.0001
Ghls: Poor	3% (31)	13% (6)	0.01
*Mental health*			
Depression	4.6% (48)	65.2% (30)	<0.0001
Type D Personality	23.3% (240)	65.2% (30)	<0.0001
Panic disorder	4.1% (41)	28.6% (12)	<0.0001
Antidepressants	5.7% (59)	21.7% (10)	0.0004
Anxiolytics	1.8% (18)	4.3% (2)	0.21

Abbreviations: Glhs (general health status), SES (socioeconomic status).

## Discussion

This is the first population-based study in a large European cohort based on cross-sectional and longitudinal data analyzing both the prevalence and new onset of depression and anxiety in subjects with AMD and investigating the association between both mental conditions and AMD.

In the present study, we did not find AMD subjects to be more often depressed than those without AMD and there was no association between AMD and depression. The prevalence of depression was 7.2% for subjects with AMD and 8.0% for subjects without AMD. Participants without AMD were more often anxious (7.0% vs. 4.2%) and AMD was associated with less anxiety after multiple logistic regression. New onset of depression and anxiety was similar in both groups and 2.6% and 3.6%, respectively for subjects with AMD and 4.5% and 4.8%, respectively for subjects without AMD.

Prior studies showed a great inconsistency in the prevalence of depression in patients with AMD. Depression prevalence varied between 2% and 44.4% in different studies^8,10,11,31–35^. Anxiety prevalence among AMD patients was reported to be as high as 10.5% in Australia^32^ and 11.7%^31^ and 30.1%^8^ in Germany.

When discussing previous findings, it is important to differentiate between case-control studies and cohort studies, as the study design might have an impact on the findings. Most case-control studies showed a higher prevalence of depression and anxiety in AMD patients than those without. Jacob *et al*. examined AMD patients’ data in the database of primary care practices in Germany and compared AMD cases to controls matched for age, sex, type of health insurance and the Charlson comorbidity index^36^. The prevalence of depression was 33.7% (vs. 27.3% in controls), and 11.7% for anxiety (vs. 8.2% for controls) and both conditions were positively associated with AMD^31^. In this study, assessment of depression and anxiety was based only on ICD codes entered by general practitioners, which may lead to a bias of results. Furthermore, visual acuity, which is known to have an impact on depression^8^, was not recorded, and adjustment for socioeconomic variables was not possible.

In a prospective, observational, multicentre study, Augustin *et al*. examined data of 360 patients from specialized retinal disease centers in Germany and found that overall prevalence was 17.9% for depression and 30.1% for anxiety. The prevalence of severe depression was reported to increase with decreasing visual acuity and depression was associated with poor visual acuity (VA), whereas anxiety was unrelated to visual acuity loss. In contrast to our study populations’ good visual acuity in both groups (mean VA in AMD: 0.1 logMar and 0.0 logMar in non-AMD), best-corrected binocular VA < 0.3 logMar in this work was retained only by 34% of the patients^8^.

Similar depression prevalence was found in further cross-sectional studies^33,34^. A study by Augustin *et al*. that included participants from a specialized retinal clinic reported that more than 50% of the patients were diagnosed with wet AMD, which is considered late AMD according to the Rotterdam Eye Study classification^17^. In contrast only 0.6% of our study population was categorized with late AMD and only 0.1% with wet AMD. The low rate of late AMD cases and the good visual acuity of our population-based cohort might explain the low depression and anxiety prevalence. Assuming the causal pathway between eye disease, visual impairment and depression/anxiety, our study population is not at elevated risk for depression and anxiety, because the overall visual acuity was good in our study cohort. It should be taken into account that a population-based design may lead to an underestimation of depression and anxiety prevalence, because the presence of depression and anxiety might prevent participants to take part in studies.

The treatment of wet AMD includes intravitreal injections, which has been reported to have an impact on patients Quality of Life^37^. As patients were recruited from specialized retinal centers, progressive AMD cases with worse VA were more often present in these studies than in our population-based cohort with good visual acuity and few cases of late AMD. Furthermore, mental health and general health scores were reported to be reduced in the AMD group by Mathew *et al*., which may additionally explain the higher depression prevalence^34^. Therefore, outcome of case-control studies should be interpreted with caution as they differ in study design, sample size (between 101 and 360 patients), study population and depression and anxiety measurement techniques, with different definitions of depression and anxiety^8,33,34^.

Findings from population-based studies were contradictory. In a Korean population-based study, participants with AMD more often reported depressive symptoms and in an Australian population-based study AMD was associated with depression, but not with anxiety^32,35^. Prevalence of early and late AMD was not considered in those studies. Both cohort studies have major limitations. The Korean study used a non-validated survey instrument for identifying depression, in contrast to the established Patient Health Questionnaire (PHQ-9) we used in our study. In this study, a single question (“Have you felt sorrow or despair that has affected your daily life for more than 2 weeks continuously during the past year?”) was provided to measure the degree of depressive symptoms, which may limit the accuracy of the responses^35^. In contrast, the PHQ-9 we applied is an established survey instrument in Germany with an high reliability and validity and is reportedly a useful screening tool for identifying symptoms of depression^19,38–40^. The Australian study was limited by self-reported AMD diagnosis, which may be biased by participants recall^32^.

Our results on prevalence and association of depression with AMD agree with the results of Jonas *et al*. in a population-based study in a rural and urban region in China^10^. Jonas *et al*. reported the prevalence of depression to be as high as 2.0% and did not confirm an association between depression and AMD. Their study population comprised 3,267 participants; socioeconomic background was examined and included in regression analysis and depressive symptoms were evaluated using a validated Chinese depression scale^41^. In line with our results and those of Jonas and colleagues, Sun *et al*. also found no association between depression and AMD in a population-based study^11^. The rate of late AMD (1.3%) was similar to that in our study and explains the contradicting results to studies recruiting patients from tertiary care hospitals, with a consequently higher rate of late AMD. Depression was assessed with a validated 10 items depression scale (Centers for Epidemiologic Studies depression scale)^42^. Extending the definition of depression and including participants using antidepressant medications did not alter the outcome. However, the results of this specific study should be interpreted in the context of one important limitation. The retinal photographs for AMD grading were taken nine years after the baseline examination, which makes an underestimation of depression in AMD likely, as cases with depression have a higher mortality as confirmed in previous studies^43,44^.

Depression was not significantly associated with AMD in the present study. We performed multivariate regression analysis, adjusting for age, sex and SES. Adjusting additionally for ocular diseases, systemic diseases, visual acuity and mental health parameter (Type D personality, loneliness, general health status) did not alter our findings. However, after adjustment for these parameters anxiety was associated with AMD and our analysis indicated that AMD diagnosis was associated with lower self-reported symptoms of anxiety. The finding that anxiety was increased in subjects without AMD in the fully adjusted model was counterintuitive as we had anticipated increased anxiety in AMD. We cannot preclude this finding to indicate that at least early stages of AMD do not affect anxiety in a significant way. A possibility could be the small numbers identified with depression (n = 78) and anxiety (n = 46) in the AMD cohort. Our analysis revealed that Type D personality, loneliness, and general health status were predictors of depression and anxiety. Our findings might suggest that the perception of having poor general health as well as social parameter and history of other mental conditions are strong risk factors for depression and anxiety. Consistent with our findings, Jivraj *et al*. reported that living with others was an independent protective factor against depressive symptoms^33^. They hypothesized that the social environment might have a possible protective role in preventing depression. The association between loneliness and depression was also reported in other previous studies^45,46^. In line with these findings, Mathew *et al*. indicated in an analysis of “causal” pathways that AMD led to depressive symptoms via reduced general health and social functioning^34^. These findings might suggest that a reduced general health and limited participation in social activities, due to reduced VA, may be the causal link between eye diseases in general and depression or anxiety. This is also supported by Augustin *et al*. who reported that self-rated depression in patients with AMD was associated with reduced VA^8^. After a direct comparison of AMD participants with and without depression and anxiety we confirm the outcomes of Jivraji, Augustin and Mathew *et al*. The direct comparison revealed that AMD-participants with depression and anxiety had a similar visual acuity, but significantly more often a co-diagnosis of Type D personality, panic disorder, anxiety and depression, respectively, a worse general health status, less social support and were more often lonely.

There are no data on incidence or new onset of depression and anxiety in AMD patients. The new onset of depression and anxiety was 2.8% and 3.7% in AMD subjects in the present cohort-study. Our findings may contribute to a better comprehension of comorbidities in persons with AMD.

The strength of the present study is the population-based prospective design, with a large sample size, standardized examinations, established mental health survey instruments and access to vast variety of potential confounders. Furthermore, we conducted different sensitivity analyses to partially overcome potential confounding factors as the overall good VA of the study population and participants under treatment for depression and anxiety at time of the study. Of course, limitations of our study have to be taken into account. It is possible that both reduced visual acuity and severe depression led to reduced participation, and indeed our study only had a small number of participants with significantly reduced visual acuity and with severe depressive symptoms. Thus, we cannot comment on depression in patients suffering from severe vision loss due to advanced AMD. To partially overcome this, we conducted a subsample analysis of participants with a visual acuity >0.3 logMar in the worse eye and the best eye and revealed similar results. However it should also be noted that the prevalence of early and late AMD in our study was in the range of other cohort studies^47^. The screening-tools applied (PHQ-9 and GAD-2) to establish new onset of depression and generalized anxiety at follow-up examination, have a limited time frame of two weeks. Therefore, it could not be assessed if participants were depressed or anxious outside the two-weeks window and we could not evaluate the timing of diagnosis with the onset of depression or anxiety. It should also be noted that no additional help was available to participants with visual impairment and disability resulting in selection bias and the measurement of participant’s corrective devices was not obtained.

In addition, we did not validate the diagnosis of AMD with optical coherence tomography findings or relate the type of AMD to treatment with intravitreal injections, which might have an impact on Quality of Life. Frequent visits with an invasive treatment and potential side effects might have a strong impact on participants wellbeing and consequently on the prevalence of depression and anxiety. Another possible confounder might be the time between AMD diagnosis and answering the PHQ-9 and GAD-2 questionnaire. It is possible that participants with a previous AMD diagnosis are more stable and have a smaller prevalence of depression and anxiety compared to those being recently diagnosed with a potential blindness causing eye disease.

In summary, this is the largest population-based cohort study analyzing the prevalence and association of depression and anxiety in subjects with AMD in a European cohort and the first to analyze new onset of depression and anxiety. Depression and anxiety were not more prevalent in subjects with AMD, and we found no association of depression with AMD.

## Supplementary information

Supplementary Tables S1 and S2

## Data Availability

The written informed consent of GHS study participants does not approve public access to the data. This concept was requested by the local data protection officer and ethics committee. Access to data at the local database in accordance with the ethics vote is offered upon request at any time. Interested researchers can make their requests to the Principal Investigators of the Gutenberg Health Study (email: info@ghs-mainz.de).
