# Supplementary material for: Prevalence and new onset of depression and anxiety among participants with AMD in a European cohort
Source: Sci Rep. 2020 Mar 16;10:4816. doi: 10.1038/s41598-020-61706-8 (PMC7075932; doi:10.1038/s41598-020-61706-8)
Supplement: Supplementary file 1 — Supplementary Tables S1 and S2 [file 41598_2020_61706_MOESM1_ESM.pdf]

Prevalence and new onset of depression and anxiety among participants with AMD in a European cohort

Jasmin Rezapour[1,2]*, Alexander K Schuster[1], Stefan Nickels[1], Christina A Korb[1], Hisham Elbaz[1,3], Tunde Peto[4,5], Matthias Michal[6], Thomas Münzel[7], Philipp S Wild[8,9,10], Jochem König[11], Karl Lackner[12], Andreas Schulz[9,] Norbert Pfeiffer[1], Manfred E Beutel[6]

1 Department of Ophthalmology, University Medical Center of the Johannes Gutenberg-University Mainz, Mainz, 55131, Germany

2 Hamilton Glaucoma Center, Shiley Eye Institute, Department of Ophthalmology, UC San Diego, La Jolla, CA 92037, United States

3 Department of Ophthalmology, Otto-von-Guericke University, Magdeburg, Germany

4 NIHR Biomedical Research Center at Moorfields Eye Hospital NHS Foundation Trust and UCL Institute of Ophthalmology, London, EC1V 9EL, United Kingdom

5 Queen's University Belfast, Centre for Public Health, Belfast, BT7 1NN, Northern Ireland, United Kingdom

6 Department of Psychosomatic Medicine and Psychotherapy, University Medical Center of the Johannes Gutenberg-University Mainz, Mainz, 55131, Germany

7 Center for Cardiology I, University Medical Center of the Johannes Gutenberg-University Mainz, Mainz, 55131, Germany

8 Preventive Cardiology and Preventive Medicine / Center for Cardiology, University Medical Center of the Johannes Gutenberg-University Mainz, Mainz, 55131, Germany

9 Center for Thrombosis and Hemostasis (CTH), University Medical Center of the Johannes Gutenberg-University Mainz, Mainz, 55131, Germany

10 German Center for Cardiovascular Research (DZHK), partner site Rhine-Main, Mainz, 55131, Germany

11 Institute for Medical Biostatistics, Epidemiology and Informatics, University Medical Center of the Johannes Gutenberg-University Mainz, Mainz, 55131, Germany

12 Institute for Clinical Chemistry and Laboratory Medicine, University Medical Center of the Johannes Gutenberg-University Mainz, Mainz, 55131, Germany

*Corresponding author: Jasmin Rezapour, jasmin.rezapour@gmail.com

Running Head: Prevalence and new onset of depression and anxiety in AMD

PHQ-9 ≥10 (N=11780)	*Model 1*		
	OR	CI	p-value
AMD	1.03	0.80 - 1.31	0.84
Sex (Women)	1.48	1.29 - 1.70	<0.0001
Age [5 years]	0.89	0.86 - 0.92	<0.0001
SES	0.93	0.92 - 0.95	<0.0001
PHQ-9 ≥10 (N=11421)	*Model 2*		
	OR	CI	p-value
AMD	0.93	0.70 - 1.23	0.62
Sex (Women)	1.36	1.15 - 1.59	0.01
Age [5 years]	0.90	0.87 - 0.94	<0.0001
SES	0.99	0.98 - 1.01	0.58
Visual acuity best eye	0.51	0.20 – 1.25	0.15
Self-reported ocular diseases	1.14	0.68 – 1.82	0.60
Arterial hypertension	0.94	0.79 – 1.12	0.48
Myocardial infarction	1.04	0.62 – 1.68	0.87
Stroke	1.46	0.85 – 2.40	0.16
Diabetes mellitus	1.05	0.79 – 1.37	0.75
COPD	0.55	0.23 – 1.32	0.18
Bronchial asthma	1.70	0.81 – 3.51	0.16
Type D personality	3.02	2.58 – 3.53	< 0.0001
Social support	0.91	0.90 - 0.93	< 0.0001
Loneliness	2.98	2.48 - 3.57	< 0.0001
General Health status (from good to poor)	3.41	3.05 - 3.83	< 0.0001

Supplementary Table S1: Associations (multivariate logistic regression analysis) of AMD with depression in the Gutenberg Health Study (GHS) and significantly associated parameters.
Abbreviations: CI (95% confidence Interval), COPD (chronic obstructive pulmonary disease, OR (Odds ratio), PHQ-9 (Patient Health Questionnaire), SES (socioeconomic status)
PHQ-9 ≥10 defined as caseness for depression
Model 1: Logistic regression analysis adjusted for age, sex, socio-economic status
Model 2*: Additionally, adjusted for systemic comorbidities, ocular diseases, visual acuity of the best eye, mental health (Type D personality, loneliness, social support) and general health status

GAD-2 ≥3 (N=11719)	*Model 1*		
	OR	CI	p-value
AMD	0.74	0.54 - 1.00	0.06
Sex (Women)	1.59	1.38 - 1.86	<0.0001
Age [5 years]	0.87	0.84 - 0.89	<0.0001
SES	0.96	0.94 - 0.98	<0.0001
GAD-2 ≥3 (N=11409)	*Model 2*		
	OR	CI	p-value
AMD	0.67	0.47 – 0.93	0.02
Sex (Women)	1.48	1.26 - 1.75	<0.0001
Age [5 years]	0.89	0.85 - 0.93	<0.0001
SES	1.01	0.99 - 1.03	0.21
Visual acuity best eye	0.47	0.17 – 1.24	0.13
Self-reported ocular diseases	0.94	0.52 – 1.60	0.84
Arterial hypertension	0.94	0.79 – 1.12	0.51
Myocardial infarction	0.93	0.50 – 1.61	0.81
Stroke	0.89	0.43 – 1.64	0.72
Diabetes mellitus	0.89	0.65 – 1.20	0.45
COPD	1.88	0.72 – 5.18	0.21
Bronchial asthma	0.81	0.32 – 1.88	0.64
Type D personality	2.79	2.37 - 3.29	< 0.0001
Social support	0.94	0.93 - 0.96	< 0.0001
Loneliness	2.36	1.94 - 2.86	< 0.0001
General Health status (from good to poor)	2.61	2.33 - 2.94	< 0.0001

Supplementary Table S2: Associations (multivariate logistic regression analysis) of AMD with generalized anxiety in the Gutenberg Health Study (GHS) and significantly associated parameters.
Abbreviations: CI (95% confidence Interval), COPD (chronic obstructive pulmonary disease, GAD-2 (Generalized Anxiety Disorder Scale), OR (Odds ratio), PHQ-9 (Patient Health Questionnaire), SES (socioeconomic status)
GAD-2 ≥3 defined as caseness for generalized anxiety
Model 1: Logistic regression analysis adjusted for age, sex, socio-economic status
Model 2*: Additionally, adjusted for systemic comorbidities, ocular diseases, visual acuity of the best eye, mental health (Type D personality, loneliness, social support) and general health status
